# Estimating the cost-effectiveness of daclatasvir + sofosbuvir versus sofosbuvir + ribavirin for patients with genotype 3 hepatitis C virus

**DOI:** 10.1186/s12962-017-0077-4

**Published:** 2017-07-21

**Authors:** Phil McEwan, Samantha Webster, Thomas Ward, Michael Brenner, Anupama Kalsekar, Yong Yuan

**Affiliations:** 1 Health Economics & Outcomes Research Ltd, 9 Oak Tree Court, Mulberry Drive, Cardiff Gate Business Park, Cardiff, CF23 8RS UK; 2 School of Human & Health Sciences, Swansea University, Swansea, UK; 3 UK HEOR, Bristol–Myers Squibb Pharmaceuticals Ltd, Uxbridge, UK; 4 World Wide Health Economics and Outcomes Research, Bristol–Myers Squibb Pharmaceuticals Ltd, Princeton, USA

**Keywords:** Hepatitis C virus, Daclatasvir, Cost-effectiveness, Sofosbuvir

## Abstract

**Background:**

As treatments for chronic hepatitis C are moving away from interferon-containing regimens, the most appropriate allocation of resources to higher cost, interferon-free, direct-acting antiviral (DAA) regimens needs to be assessed. Hepatitis C virus (HCV) genotype 3 is associated with faster disease progression and has fewer treatment options, historically, than other HCV genotypes. This analysis aims to estimate the comparative cost-effectiveness of two recently licenced interferon-free regimens for the treatment of HCV genotype 3.

**Methods:**

Utilising a published Markov model and results of a matching-adjusted indirect comparison of recently published clinical trial data (ALLY-3 and VALENCE, respectively), 12 weeks of treatment with daclatasvir + sofosbuvir (DCV + SOF) was compared to 24 weeks of treatment with sofosbuvir + ribavirin (SOF + RBV). UK-specific model inputs were used to inform a cost-utility analysis of these regimens.

**Results:**

In the base case analysis, DCV + SOF was found to be dominant over SOF + RBV in treatment-naïve patients, patients that had previously been treated, and patients that are intolerant to, or ineligible for, interferon-containing regimens. Given the low rates of treatment currently observed in the UK, DCV + SOF was also compared to no treatment in the interferon-ineligible/intolerant patients, and may be considered cost-effective with an incremental cost-effectiveness ratio of £8817.

**Conclusions:**

When compared to 24 weeks of SOF + RBV, 12 weeks of treatment with DCV + SOF results in improved quality of life and reduced total costs, and therefore is likely to represent significant clinical and economic value as a treatment option for genotype 3 HCV infection.

**Electronic supplementary material:**

The online version of this article (doi:10.1186/s12962-017-0077-4) contains supplementary material, which is available to authorized users.

## Background

Chronic hepatitis C is a progressive disease of the liver, affecting an estimated 214,000 people in the UK [1]. Chronic hepatitis C often results in the development of cirrhosis (i.e. permanent scarring), which can lead to end-stage liver disease (ESLD), and is a major risk factor for hepatocellular carcinoma (HCC) [2–5]. Rates of hepatitis C-related ESLD and HCC are rising, resulting in increased demand for transplant and increased rates of mortality [1].

Hepatitis C virus (HCV) subtypes 1 and 3 predominate in the UK [1]; however, HCV genotype 3 is associated with increased rate of progression to cirrhosis and liver decompensation, increased oncogenesis, and is accepted to be harder to treat than other genotypes, partially due to the limited number of therapy options [6–12]. Genotype 3 represents a significant proportion (~43%) of the infected UK population [1], hence prevention of ESLD, transplant and death in this large pool of patients is vital and is likely to be of value from both a public health, payer and individual perspective.

The treatment of choice, from both a clinical and patient perspective, is rapidly shifting from regimens that are interferon-based, due to efficacy (defined by a sustained virologic response [SVR] to treatment) and tolerability issues, to direct-acting antiviral (DAA) regimens, with high efficacy and good safety profiles. As such, payers are faced with difficult decisions regarding the most appropriate allocation of resources to these treatments, given their relatively high costs. Three interferon-free regimens are currently available in the UK for the treatment of HCV genotype 3: sofosbuvir in combination with ribavirin (SOF + RBV), sofosbuvir in combination with ledipasvir (SOF + LDV) and daclatasvir in combination with sofosbuvir (DCV + SOF). Current clinical guidelines published by the European Association for the Study of the Liver (EASL) recommend the use of DCV + SOF in patients with HCV genotype 3, with treatment prioritised in patients with advanced fibrosis (i.e. those with a METAVIR score of F3–F4) [13].

The objective of this study was to investigate the cost-effectiveness of DCV + SOF versus no treatment, given that treatment uptake rates are currently around 3% in the UK [1], and versus the alternative interferon-free regimen, SOF + RBV.

## Methods

### Model

A previously published and validated chronic hepatitis C Markov model was used to estimate the cost-effectiveness of interferon-free therapy regimens for treatment of HCV genotype 3 patients [14–16]. The model predicts the natural history of chronic hepatitis C through METAVIR fibrosis stages F0–F4 and on to ESLD complications and death (Additional file 1: Figure S1). A cohort of patients progress through METAVIR fibrosis stages F0–F4 via dynamic transition rates from a meta-regression analysis of a multi-country, multi-centre study using data from 33,121 individuals chronically infected with HCV [17]. Progression to ESLD and HCV-related death were modelled using previously published static transition rates, whilst non-HCV-related mortality was estimated through the incorporation of published UK-specific life tables [18–20]. Disease transition rates are summarised in Table 1. To simulate the increased rate of disease progression observed amongst HCV genotype 3 patients, published transition rate multipliers were applied to the rates of disease progression previously described [12]. In those patients failing to achieve SVR, disease progression continues from the stage in which therapy commenced. Half-cycle corrections were applied to the models estimates of disease progression.

**Table 1:** Disease transition rates

Transition	Mean	SE	Sources
F0 to F1	0.084	NA	[17]
F1 to F2	0.092	NA	
F2 to F3	0.145	NA	
F3 to F4	0.116	NA	
F4 (compensated cirrhosis) to decompensated cirrhosis	0.039	0.010	[20]
F4 (compensated cirrhosis) to HCC	0.014	0.010	
Decompensated cirrhosis to HCC	0.014	0.010	
Decompensated cirrhosis to liver transplant	0.030	0.012	
Decompensated cirrhosis to death	0.130	0.010	
HCC to liver transplant	0.030	0.012	
HCC to death	0.430	0.030	
Liver transplant (year 1) to death	0.210	0.046	
Liver transplant (year 2+) to death	0.057	0.012	

*HCC* hepatocellular carcinoma, *NA* not applicable, *SE* standard error

Each health state within the model is associated with a particular cost and health utility estimate, as presented in Table 2. All values utilised within the model are consistent with a published systematic literature review, which have been used extensively in previous economic evaluations [21–23]. All costs were inflated to 2013 values using the Hospital and Community Health Services index, where required [24]. Costs and health utility estimates were discounted annually at a rate of 3.5%, in line with UK guidelines [25]. Total costs and quality-adjusted life years (QALYs) associated with each treatment regimen are accumulated over the modelled time horizon and used to predict the cost-effectiveness of each comparison.

**Table 2:** Summary of health state cost and utility inputs

Health state	Cost (2012/13 £)	Utility	Source
	Mean (SE)	Mean (SE)	
Fibrosis stages			
F0	177.47 (35.01)	0.77 (0.015)	[21]
F1	177.47 (35.01)	0.77 (0.015)	
F2	922.08 (97.82)	0.66 (0.031)	
F3	922.08 (97.82)	0.66 (0.031)	
F4 (compensated cirrhosis)	1463.50 (297.45)	0.55 (0.054)	
SVR			
SVR from F0 to F1	333.08 (62.05)	0.82 (0.043)	[21]
SVR from F2 to F3	922.08 (97.74)	0.72 (0.048)	
SVR from F4 (compensated cirrhosis)	1463.50 (288.07)	0.72 (0.048)^a^	
ESLD			
Decompensated cirrhosis	11,728.61 (1954.09)	0.45 (0.031)	[21]
HCC	10,451.58 (2456.09)	0.45 (0.031)	
Liver transplant (transplant cost)	35,147.26 (3709.93)	NA	
Liver transplant (cost of care: initial year)	12,163.29 (3133.55)	0.45 (0.031)	
Liver transplant (subsequent years)	1781.15 (456.57)	0.67 (0.066)	

*HCC* hepatocellular carcinoma, *NA* not applicable, *SE* standard error, *SVR* sustained virologic response, *ESLD *end stage liver disease

^a^Assumption, based on SVR from F2 to F3

For this analysis, since there is a paucity of robust epidemiology data, patients within the modelled cohort were assumed to be evenly distributed across fibrosis stages F0–F4 at initiation. A mean age of 50 years was applied, of which 67% were male, according to UK estimates [26–28]. The cohort was modelled over a lifetime horizon, assuming a maximum age of 100 years. It was assumed that the progression of disease is halted following SVR, regardless of fibrosis stage at therapy initiation.

### Treatment

Treatment with DCV + SOF was compared to SOF + RBV in three patient populations; treatment-naïve, treatment-experienced and interferon-ineligible/intolerant. To provide economic context for the potential consideration of not treating patients who may be more difficult to treat, an additional comparison against “no treatment” was undertaken in interferon-ineligible/intolerant patients. As direct comparative data were not available for the treatment regimens under investigation, an indirect comparison of efficacy and safety observed in the ALLY-3 (NCT02032901) and VALENCE (NCT01682720) phase III clinical trials of DCV + SOF and SOF + RBV, respectively, was carried out via the matching-adjusted indirect comparison (MAIC) method [29, 30]. MAIC data were available for DCV + SOF and SOF + RBV in the treatment-naïve and treatment-experienced patient populations [31]. However, as no data specific to the interferon-ineligible/intolerant population were identified for the SOF + RBV regimen, it was assumed that the efficacy in this subgroup was equivalent to the SVR in the pooled naïve and experienced subgroups since this subgroup would inherently comprise patients that have not received treatment (due to contraindications) or have received treatment, but discontinued (due to tolerability). Treatment-related model inputs are detailed in Table 3.

**Table 3:** Treatment-related model inputs

Regimen	Duration (weeks)	Acquisition cost		SVR			Disutility	
		Per week (£)	Source	Population	%	Source	Mean	Source
DCV + SOF	12	4958.37	Monthly index of medical specialities	Treatment-naïve	96.4	MAIC [30]	0.035	Estimated from [37]
				Treatment-experienced	83.2			
				Interferon-ineligible/intolerant	88.8^a^			
SOF + RBV	24	2982.19	Monthly index of medical specialities	Treatment-naïve	94.3	MAIC [30]	0.048	Estimated from [38]
				Treatment-experienced	78.6			
				Interferon-ineligible/intolerant	85.2^a^			
No treatment	NA	0	NA	Interferon-ineligible/intolerant	0	Assumed	0	NA

*DCV* daclatasvir, *NA* not applicable, *MAIC* matching-adjusted indirect comparison, *RBV* ribavirin, *SOF* sofosbuvir, *SVR* sustained virologic response

^a^Assumed, based on pooled treatment-naïve and treatment-experienced subgroups

Unit costs of treatments were sourced from the Monthly Index of Medical Specialities (MIMS) and regimen durations were modelled according to EASL guidelines and trials used [2, 29, 30]. Due to their relative infrequency, costs relating to treatment-related adverse events and discontinuations were not modelled; however, regimen-specific disutilities were applied for the duration of therapy (Table 3). As data relating to the costs of monitoring patients receiving new DAA therapies are not yet available, monitoring costs consistent with previous health technology assessments of pegylated interferon-alpha-based regimens were utilised [20].

### Analysis

The analysis was performed from the perspective of the UK NHS and personal social services. Base case analysis compared total costs and QALYs of each regimen and estimated the incremental cost-effectiveness ratio (ICER) for each comparison.

One-way sensitivity analyses were undertaken in order to assess the impact of individual parameters on cost-effectiveness, whilst probabilistic sensitivity analysis (PSA) was undertaken to assess the uncertainty surrounding model input parameters. Static transition rates, proportions and utilities were sampled from beta distributions, age and coefficients of the dynamic transition rates from normal distributions and costs from gamma distributions.

To address uncertainty surrounding rates of SVR, an exploratory analysis was undertaken to estimate the minimum rate of SVR of the DCV + SOF regimen such that it would remain cost-effective at the £20,000/QALY threshold [32].

## Results

### Base case analysis

The base case analysis demonstrates that DCV + SOF is cost-effective against SOF + RBV in all comparisons tested: treating with DCV + SOF was predicted to result in total cost savings of £12,647, £13,426 and £12,305 in treatment-naïve, treatment-experienced and interferon-ineligible/intolerant populations respectively. Predicted QALY-gains were 0.118, 0.219 and 0.165 respectively, resulting in DCV + SOF dominating SOF + RBV in all comparisons. When comparing to no treatment in the interferon-ineligible/intolerant population, DCV + SOF was predicted to give rise to an incremental cost of £32,476, with a QALY gain of 3.683. The associated ICER estimate was £8817, which is cost-effective at the £20,000/QALY threshold (Table 4).

**Table 4:** Results: Base case cost-effectiveness, PSA and exploratory analyses

Treatment population	Intervention			Comparator			Incremental results		ICER (£/QALY)^a^	Probability cost-effective (%)^a^	Lowest SVR at which DCV + SOF remains cost-effective (%)^a^
	Regimen	Total cost (£)	QALYs	Regimen	Total cost (£)	QALYs	Total cost (£)	QALYs			
Treatment naïve	DCV + SOF	62,127	13.233	SOF + RBV	74,774	13.115	−12,647	0.118	DCV + SOF dominant	100	83.3
Treatment experienced		66,241	12.685	SOF + RBV	79,667	12.466	−13,426	0.219	DCV + SOF dominant	100	67.6
Interferon-ineligible/intolerant		64,436	12.926	SOF + RBV	76,740	12.761	−12,305	0.165	DCV + SOF dominant	100	75.2
				No treatment	31,960	9.242	32,476	3.683	£8817	100	52.8

*DCV* daclatasvir, *ICER* incremental cost-effectiveness ratio, *QALY* quality-adjusted life year, *RBV* ribavirin, *SOF* sofosbuvir, *SVR* sustained virologic response

^a^Cost-effectiveness threshold: £20,000–£30,000/QALY

### Sensitivity analyses

**Fig. 1:**
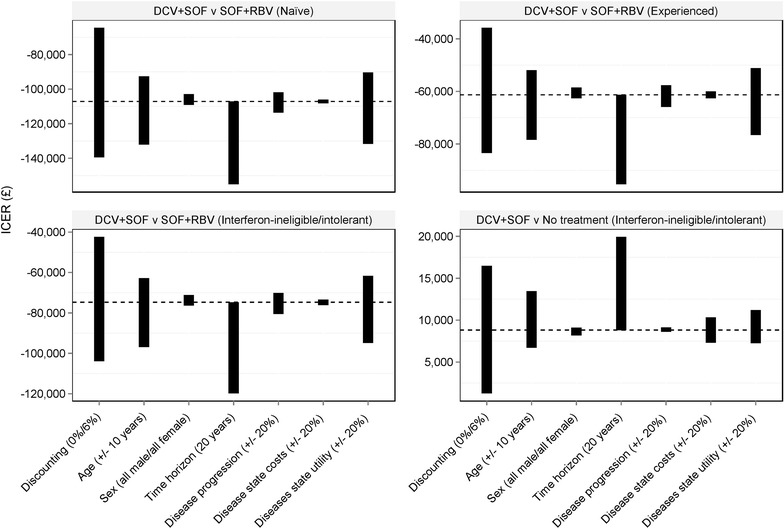
Univariate sensitivity analysis. *DCV* daclatasvir, *RBV* ribavirin, *SOF* sofosbuvir

Univariate sensitivity analyses demonstrated that the cost-effectiveness is most sensitive to assumptions surrounding discounting, patient age and model time horizon (Fig. 1). Using a model time horizon of 20 years decreases expected cost-effectiveness; however, no conclusions changed. Increasing rates of annual discounting resulted in reduced cost-effectiveness, whilst decreasing the rate resulted in improved cost-effectiveness. A similar trend was observed when increasing and decreasing age.

PSA demonstrated that all comparisons have a 100% probability of cost-effectiveness when parameter uncertainty was taken into consideration, with all individual PSA simulations resulting in cost-effective results, across all scenarios.

### Exploratory efficacy analysis

Analysis undertaken to estimate the possible reduction in DCV + SOF SVR rates at which the regimen is no longer expected to be cost-effective predicted that the SVR rate of DCV + SOF could be reduced by 13.1%, 15.6% and 13.6%, amongst treatment-naïve, treatment-experienced and interferon-ineligible/intolerant patients respectively before the regimen is no longer expected to be cost-effective against SOF + RBV at the lower cost-effectiveness threshold of £20,000/QALY. Similarly, it was predicted that the SVR of DCV + SOF could be reduced by 26.0%, when compared to no treatment, before it is no longer expected to be cost-effective (Table 4).

## Discussion

Since an estimated 92,000 individuals, almost half the HCV-infected population, have genotype 3 in the UK, there is a clear need to provide clinical and cost-effective treatment options for this considerable pool of patients [1]. Presented is the first cost-effectiveness analysis of two interferon-free regimens for the treatment of HCV genotype 3 in a UK context. The results of this analysis demonstrate the significant value that is expected to be realised by the use of 12 weeks of DCV + SOF over 24 weeks of SOF + RBV or no treatment. These conclusions are consistent with a published cost-effectiveness analysis in the Italian setting [33].

Considering the cost savings estimated, a lower overall budget impact is expected via the adoption of DCV + SOF over SOF + RBV. Cost-savings and QALY gains for the DCV + SOF regimen are largely driven by the shorter treatment duration, higher SVRs and the resulting avoidance of high cost ESLD complications.

These are important findings, given that there are currently few treatment options for HCV genotype 3, especially considering the high proportion of patients that cannot tolerate, are not recommended to receive or are unwilling to take an interferon-containing regimen, estimated at 67.0–83.5% of infected patients [34, 35]. Furthermore, EASL clinical guidelines recommend DCV + SOF in patients with HCV genotype 3 due to the suboptimal efficacy of the SOF + RBV regimen [13].

Given the rapidly developing nature of the field, challenges exist in comparing novel regimens. To inform this analysis, an indirect comparison was performed in an attempt to eliminate bias that may have been observed via a naïve comparison of the raw trial data; however, in the absence of direct head-to-head data, some uncertainty remains. Furthermore, due to the unavailability of data specifically for interferon-ineligible/intolerant patients for either DCV + SOF or SOF + RBV, an assumed SVR, taking the entire patient population (treatment-naïve and -experienced patients) of the trials into consideration, has been used. It is uncertain whether this will under- or over-estimate costs and QALYs in this population; however, a consistent approach has been applied to both regimens. Despite uncertainties in the data, SVR threshold analysis gives confidence in the level by which the SVR of DCV + SOF can fall to no longer be cost effective versus both SOF + RBV and no treatment (8.5–28.6% reduction in SVR, depending on the scenario).

It has previously been demonstrated that disease progression in patients with genotype 3 HCV infection occurs faster than that of other genotypes [6–12]. However, with respect to the modelling of HCV genotype 3, genotype-specific disease state transition rates are unavailable, with economic models relying on the use of transition rate multipliers applied to disease progression rates of a genotype 1 population. Therefore, further research into the patterns of disease progression of HCV genotype 3 patients may be warranted to more accurately predict the disease progression of such patients. In addition, it was assumed that the progression of disease is halted following SVR, regardless of fibrosis stage at therapy initiation. There is some evidence to suggest that disease may progress after SVR [36, 37]; however, this rate is very low (~1% progress to cirrhosis) and is not expected to meaningfully impact cost-effectiveness results [37].

DCV + SOF provides a short, safe and effective treatment option for HCV genotype 3 patients. Considering that the majority of infections occur via injecting drug use, there is evidence to suggest that the introduction of novel DAA regimens offers the opportunity to decrease onward transmission of HCV [38], considering their improved efficacy and tolerability, with reduced time on treatment, compared to historical treatments. This means that a conventional cost-effectiveness evaluation, as presented here, is likely to underestimate the benefit, and therefore value, of treatment. Furthermore, this analysis assumed equal distribution of patients across fibrosis stages F0–F4; given the findings of previous economic analyses in chronic hepatitis C, there is likely to be higher value in taking a targeted approach to treatment, i.e. prioritising treatment in those with most advanced disease (≥F3), before the development of ESLD complications.

In this continually evolving field, there is emerging preference for interferon-free regimens from both the patient and clinician perspective. Currently, as well as DCV + SOF and SOF + RBV, a third interferon-free regimen (SOF + LDV) is available in the UK for treating HCV genotype 3; this regimen has recently received marketing authorisation; however, data for this regimen are very limited in HCV genotype 3 and are restricted to a regimen duration that is not within the marketing authorisation [39]. Therefore, a reliable comparative analysis could not be undertaken. Future analyses should incorporate this regimen, when more data are available. However, it should be noted that recent clinical guidelines recommend only the DCV + SOF regimen in patients with HCV genotype 3, as LDV has been demonstrated to be considerably less potent against genotype 3 than DCV in vitro [13].

## Conclusions

This is the first empirical analysis of contemporary clinical data describing the comparative cost-effectiveness of DCV + SOF versus SOF + RBV and no treatment in patients with HCV genotype 3. DCV + SOF was found to be dominant against SOF + RBV at established norms among treatment-naive and -experienced patients, and patients who are interferon-intolerant or ineligible. Results were robust across alternative values for key input parameters.

## Additional file

**Additional file 1: Figure S1.** Schematic of the cohort-based Markov simulation model.

## Abbreviations

DAA: direct-acting antiviral
HCV: hepatitis C virus
DCV: daclatasvir
SOF: sofosbuvir
RBV: ribavirin
ESLD: end-stage liver disease
HCC: hepatocellular carcinoma
SVR: sustained virologic response
EASL: European Association for the Study of the Liver
LDV: ledipasvir
QALY: quality-adjusted life year
MAIC: matching-adjusted indirect comparison
ICER: incremental cost-effectiveness ratio
PSA: probabilistic sensitivity analysis
MIMS: Monthly Index of Medical Specialities
