# Supplementary figures and images for: Estimating the cost-effectiveness of daclatasvir + sofosbuvir versus sofosbuvir + ribavirin for patients with genotype 3 hepatitis C virus
Source: Cost Eff Resour Alloc. 2017 Jul 21;15:15. doi: 10.1186/s12962-017-0077-4 (PMC5521139; doi:10.1186/s12962-017-0077-4)

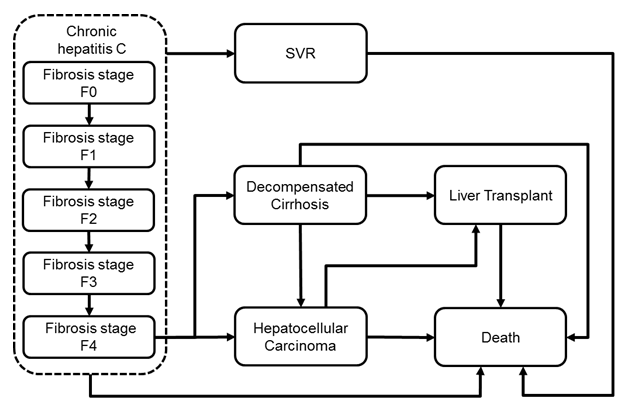

Supplement: Supplementary file 1 — Additional file 1: Figure S1. Schematic of the cohort-based Markov simulation model. [file 12962_2017_77_MOESM1_ESM.png]
